# The Potential of Focal Muscle Vibration Therapy in the Management of Parkinson’s Disease: A Systematic Review

**DOI:** 10.3390/jcm14217472

**Published:** 2025-10-22

**Authors:** Daniel Rafti, Andreea-Bianca Uzun, Lavinia Bodeanu, Liliana-Elena Stanciu, Marius-Nicolae Popescu, Madalina-Gabriela Iliescu

**Affiliations:** 1 Vraja Marii Balnear Complex, Block E2, Mircea cel Bătrân Street, 900642 Constanța, Romania; 2 Faculty of Medicine, Doctoral School, “Ovidius” University of Constanta, 1 University Alley, Campus-Corp B, 900470 Constanta, Romania; bianca.uzun@365.univ-ovidius.ro; 3 Department of Physical Medicine and Rehabilitation, Balneal and Rehabilitation Sanatorium of Techirghiol, Victor Climescu Street 34–40, Techirghiol, 906100 Constanta, Romania; 4 Department of Rehabilitation, University of Medicine and Pharmacy Carol Davila, 8 Eroii Sanitari Blvd, 050474 Bucharest, Romania

**Keywords:** Parkinson’s disease, Parkinsonian disorders, focal muscle vibration therapy, medical rehabilitation, neurorehabilitation, vibration therapy

## Abstract

**Background and Objectives:** Parkinson’s disease is the second most common neurodegenerative disorder after Alzheimer’s disease, and its incidence increases with age, being particularly high in people over 70 years of age. For patients with this condition, medical rehabilitation can have a profound impact, helping to improve mobility, preserve functional autonomy, and enhance quality of life. Focal vibration stimulation is a promising, well-tolerated, and easy-to-apply method with potential to facilitate motor activity and support the motor learning process, making it also useful in gait reeducation for patients with various neurological conditions. This systematic review aims to analyze the existing scientific evidence on the effectiveness of focal muscle vibration therapy in managing symptoms of Parkinson’s disease. **Materials and Methods***:* This systematic review of the literature was conducted in accordance with the Preferred Reporting Items for Systematic Reviews and Meta-Analyses (PRISMA) guidelines, and the protocol was registered in PROSPERO under the protocol registration number CRD420251120737. Searches were conducted in five databases (PubMed, PEDro, ScienceDirect, Cochrane Library, Web of Science). The selection criteria targeted original clinical studies, published in English between 2010 and the present, that investigated focal muscle vibration therapy in patients with Parkinson’s disease and were fully available, excluding review papers, meta-analyses, books, and articles inaccessible in full text. Version 2 of the Cochrane risk-of-bias tool for randomized trials (RoB 2) was used to assess the quality of the included studies. **Results:** The results of the studies were interpreted individually for each study, and the main information was synthesized in a comparative table to facilitate analysis. The final analysis included five studies that investigated the effects of focal muscle vibration in patients with Parkinson’s disease. The results suggest that this form of stimulation may offer benefits for patients with gait disorders, improving balance and stability. Among the study’s limitations are the small number of included articles (n = 5) and the restriction to English-language publications, which may limit the applicability of the results. **Conclusions:** Given the promising results, focal muscle vibration therapy could represent a useful option in the management of Parkinson’s disease. Integrating this method into rehabilitation plans could bring significant functional benefits, but further studies are needed to confirm its long-term effectiveness and to establish standardized application protocols. No external funding was received for the conduct of this review.

## 1. Introduction

Parkinson’s disease (PD) is the second most common neurodegenerative disorder after Alzheimer’s disease (AD), and its incidence increases with age, being particularly high in people over 70 years of age [1]. In Europe, the prevalence is estimated to range from 108 to 257 cases per 100,000 population, with an annual incidence of between 11 and 19 cases per 100,000 people [2].

Numerous risk factors are well documented for the development of PD, including age, sex, ethnicity, rapid eye movement sleep disorder, high dairy consumption, traumatic brain injury, and exposure to pesticides or herbicides [1,2]. Although the exact cause of the disease is unknown in most cases, various genetic mutations have been identified that contribute to its occurrence [2]. On the other hand, smoking, caffeine consumption, and regular physical activity have been shown to have a protective role against the development of the disease [1].

People with PD exhibit specific motor symptoms, including tremors, muscle rigidity, slow movements (bradykinesia), and loss of postural balance. In addition, there are non-motor symptoms, such as memory and cognition disorders (dementia), urinary problems, sleep difficulties, and low blood pressure when changing position (orthostatic hypotension). Because the disease can resemble other neurological conditions, careful adherence to the diagnostic criteria is crucial for accurate assessment [3,4].

Treatment of PD is mainly symptomatic and aims to improve motor disorders (such as tremor, rigidity, or bradykinesia) and non-motor symptoms (such as constipation) [5]. It focuses mainly on the administration of drugs that act symptomatically by regulating neurotransmitters. Dopaminergic replacement therapy is well established, and levodopa remains the gold standard treatment for managing the symptoms of the disease [6]. Rehabilitation interventions and physical activity play a complementary role in the therapeutic regimen, alongside specific medication. In cases where complications arise—such as worsening symptoms, loss of treatment efficacy between doses (“break periods”), medication-refractory tremor, or the appearance of dyskinesias—advanced therapies, such as deep brain stimulation, may be recommended [5,7].

Focal muscle vibration (FMV) is a modern therapeutic method used to support the improvement of balance and motor control in various neurological conditions [8]. This technique consists of applying a vibratory stimulus, characterized by high frequency and low amplitude, directly to a specific muscle using a mechanical device. Among the advantages are improved patient compliance, long-lasting effects, stimulation of muscle mass growth, enhanced blood circulation, increased bone density, reduced joint discomfort, and lower back pain [9].

Research has focused on investigating the effects of focal vibration stimulation on different levels of the central nervous system and on understanding the pathophysiological mechanisms associated with neurological disorders [10,11]. The potential benefits of focal vibration in the context of neurorehabilitation have also been analyzed [12]. This form of stimulation has been proven to be well-tolerated, effective, and easy to apply. It is considered a promising option for reducing spasticity, facilitating motor activity, and supporting the motor learning process during functional activities, including gait reeducation, regardless of the cause of the neurological condition [12].

Focal mechanical vibration therapy stimulates the activation of neuromuscular receptors (muscle spindles and Golgi tendon organs), which transmit impulses to the central nervous system. These mechanical vibrations can stimulate neuroplasticity, an essential process in the recovery of patients with PD. By influencing excitability in both the spinal cord and the cerebral cortex, this therapy helps increase motor performance, contributing to the reduction in spasticity and improving movement control [13].

The existing literature on FMV therapy in patients with PD lacks a clear synthesis. This review was conducted to centralize the evidence, highlight gaps, and guide both clinical practice and future research. Our literature search did not identify any review or meta-analysis dedicated exclusively to FMV in patients with PD. Therefore, this review provides an up-to-date and specific synthesis, integrating the most recent clinical studies and highlighting optimal parameters, while providing a critical assessment of the therapy’s efficacy and identifying existing research gaps, which supports the originality and relevance of the paper.

This systematic review aims to analyze the existing scientific evidence on the effectiveness of FMV therapy in managing symptoms of PD, like gait, balance, and posture disturbances.

## 2. Materials and Methods

This systematic review was developed following the “Preferred Reporting Items for Systematic Reviews and Meta-Analysis”—“PRISMA”, which is internationally recognized for its contribution to standardizing the reporting of studies of this type. These guidelines facilitate a clear and transparent presentation of the research motivation, the method used, and the results obtained [14].

Additionally, the protocol used for this review was submitted to PROSPERO (International Prospective Register of Systematic Reviews) under protocol registration number CRD420251120737 [15].

### 2.1. The Search Strategy

The systematic review was conducted based on a comprehensive analysis of the specialized literature, aiming to identify, select, and critically evaluate the most relevant studies published in the field.

This was achieved through an extensive search across five databases, which was completed as of August 2025. For each database, we used the advanced search functions available. To optimize selection, filters were applied to restrict the publication period to 2010–2025, including only studies in English, with full text freely available, and focused on human subjects, while excluding systematic reviews, meta-analyses, books, book chapters, editorials, conference abstracts, and notes.

The PRISMA method was used for article selection, with the articles being sourced from several relevant international databases. Among these databases, we list: PubMed, Physiotherapy Evidence Database (PEDro), ScienceDirect, Cochrane Library, and Web of Science. The search strategy employed a predefined set of keywords, as outlined in Table 1.

To efficiently manage and organize the identified articles, the Zotero software (version 7.0.24) [16] was utilized to facilitate the rapid removal of duplicates and to manage and organize the articles. Initial selection of studies was based on title and abstract, applying clear inclusion and exclusion criteria. Subsequently, eligible articles were assessed in detail by full reading, as part of the systematic review process. Extracted data included study design, sample size, intervention characteristics, and main outcomes.

For each outcome, the effects were presented in the studies reported by the included indicators, depending on how the original authorities expressed the data.

### 2.2. The Study Selection

Article selection was carried out independently by two reviewers (by authors D.R. and A.-B. U), who applied predefined inclusion and exclusion criteria. The steps of the process included an initial screening of titles and abstracts, followed by a comprehensive assessment of the studies deemed eligible for further evaluation. In the event of differences of opinion between reviewers, these were resolved through discussion, and a common agreement was reached.

*The inclusion criteria* were the following:

Original studies (randomized controlled trials, case–control studies, cohort studies, observational studies, trials);

PD diagnosis;

Studies that used focal muscle vibration therapy;

Articles written in English;

Year of publication between 2010 and present;

Free full-text;

Articles that fall into the category of articles based on scientific evidence.

*The exclusion criteria* were the following:

Systematic reviews, meta-analyses, books, book chapters, editorials, conference abstracts, and notes;

Patients with another medical condition, besides PD;

Other therapies used, apart from focal muscle vibration therapy;

Studies published before 2010;

Studies published in languages other than English;

Studies that are not available in full text;

Animal studies or preclinical studies without clinical validation.

### 2.3. Data Extraction

Data extraction was performed independently by three reviewers using a standardized data extraction form. Any discrepancies between reviewers were resolved through discussion and consensus.

For all outcomes, effect sizes were summarized using the reported measures, such as percentages, mean values, or changes from baseline values, as provided in the original studies. Studies were assessed for inclusion in each synthesis by tabulating their key characteristics, including population, intervention, comparator, and outcomes. Only studies whose characteristics met the predefined criteria for a particular synthesis were included. Data from included studies were standardized for synthesis and presentation.

Although several studies initially appeared to meet the inclusion criteria, but were subsequently excluded. For example, some studies did not report relevant outcomes, used different intervention protocols, lacked a placebo arm, and others had insufficient follow-up.

### 2.4. The PICO Question

The included studies were selected based on their relevance to the main question of this systematic review: “In patients diagnosed with PD, does focal muscle vibration therapy have a more beneficial effect on symptomatology (motor symptoms, functionality, quality of life, and disability) compared to placebo intervention?”. This question was formulated according to the PICO structure, having the following components:

- P (Population): People diagnosed with PD;
- I (Intervention): Focal muscle vibration therapy;
- C (Comparison): Compared to placebo;
- O (Outcome): Improvement in symptoms of patients included in the study (effects of vibration therapy on motor/functional recovery and disability reduction).

To facilitate understanding of the inclusion and exclusion criteria, we structured a summary table using the PICOS (Population, Intervention, Comparison, Outcomes, Study design) framework, thus providing a clear and transparent presentation of the selection of studies included in this systematic review (Table 2).

### 2.5. Bias Assessment

Two independent reviewers evaluated the study’s risk of bias by applying Version 2 of the Cochrane risk-of-bias tool for randomized trials (RoB 2) [17], a recognized and validated instrument in the specialized literature. The updated Cochrane RoB tool for randomized trials (RoB 2), released in 2019, was developed to overcome the limitations of the original tool published in 2008 and to improve the consistency and reliability of bias assessments [18].

RoB 2 is the recommended tool for assessing the risk of bias in randomized trials included in Cochrane Reviews. RoB 2 is organized into a fixed set of bias domains, each focusing on different aspects of trial design, conduct, and reporting [19].

Disagreements were addressed through dialogue or with the involvement of a third reviewer.

Risk of bias assessment is a crucial component of systematic review methodology [18]. The RoB 2 tool evaluates the risk of bias across five domains: D1: The randomization process; D2: Deviations from intended interventions; D3: Missing outcome data; D4: Outcome measurement, and D5: The selection of the reported result [20]. A proposed judgment about the risk of bias for each domain is generated by an algorithm based on responses to signaling questions. The judgment can be classified as “Low risk”, “High risk”, or “Some concerns” [19]. The overall risk-of-bias judgment for a specific outcome is classified as low when all domains show low risk; it is rated as some concerns if at least one domain raises doubts but none are at high risk; and it is considered high risk either when any domain is judged to have high risk or when multiple domains with some concerns collectively reduce confidence in the result [21].

The RoB 2 tool uses the following colors to indicate risk of bias: Green: Risk of bias is low; Yellow: There are some concerns about the risk of bias; Red: There is a high risk of bias [19].

### 2.6. Findings and Certainty of Evidence (GRADE Assessment)

In addition to using the RoB 2 tool to assess the risk of bias of the included studies, we also applied the Grading of Recommendations Assessment, Development and Evaluation (GRADE) methodology. This system provides a transparent and systematic framework for assessing the certainty of the evidence and the strength of the conclusions of studies [22]. The integration of GRADE is important because it not only allows for the identification of potential sources of bias at the level of individual studies, but also provides an overall assessment of the rigor and confidence in the body of evidence analyzed, an essential aspect for a review article [23].

## 3. Results

**Figure 1:**
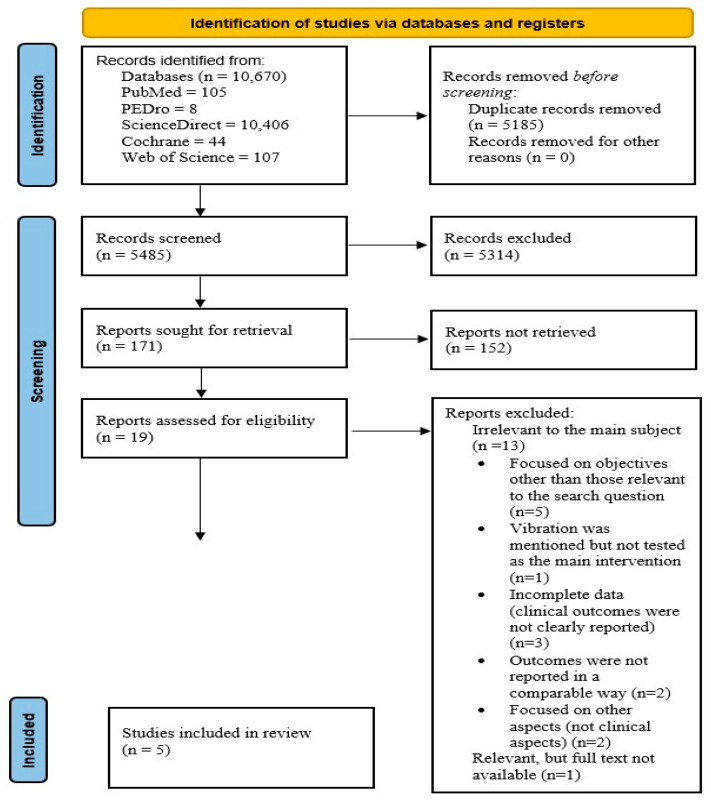
Adapted PRISMA flow diagram.

A total of 10,670 studies were initially identified, as shown in Table 1. Among the databases explored, ScienceDirect generated the largest number of results (10,406), followed by Web of Science, which returned 107 articles, and PubMed with 105 articles. The Cochrane Library contributed 44 articles, while PEDro generated a smaller number of articles (8). These identified studies formed the basis for the subsequent screening and selection process. After eliminating duplicates, 5485 articles remained. Further screening of their titles and abstracts led to the exclusion of 5314 studies. Then, a full-text assessment was performed for the remaining 171 articles, which led to the inclusion of 19 studies. Of these, 13 studies were excluded for the following reasons: focused on objectives other than those relevant to the search question (n = 5), vibration was mentioned but not tested as the main intervention (n = 1), incomplete data (clinical outcomes were not clearly reported) (n = 3), outcomes were not reported in a comparable way (n = 2), focused on other aspects (not clinical aspects) (n = 2), relevant article, but full text not available (n = 1). In the final stage, five studies remained for analysis (Figure 1).

### 3.1. The Included Studies

Following the eligibility assessment, 5 studies were selected for inclusion in this analysis (Figure 1).

The studies included in this analysis are systematically organized in Table 3, which presents key details such as author name, year of publication, study design, sample size, and references.

### 3.2. Risk of Bias

According to the Rob 2 tool, of the five studies analyzed, two [26,28] did not raise concerns, as they were assessed as having a low risk of bias in all areas. The other three studies [24,25,27] raised concerns in one of the areas (randomization or outcome selection), which led to an overall assessment of some concerns, but without significantly affecting the overall validity of the results.

**Figure 2:**
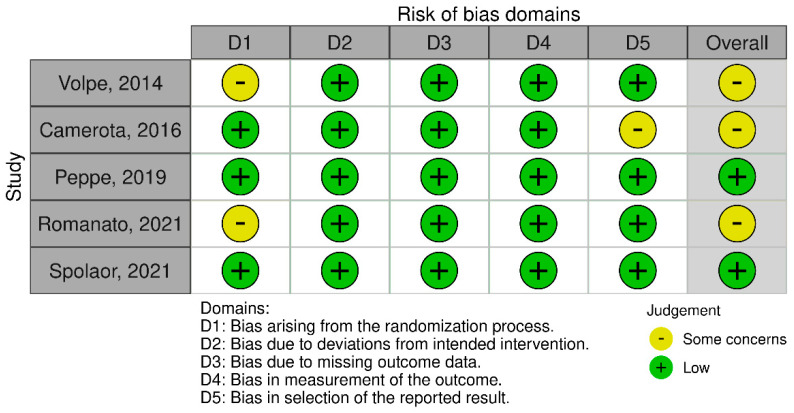
The risk of bias assessment using the RoB 2 tool for the included studies [24,25,26,27,28].

**Figure 3:**
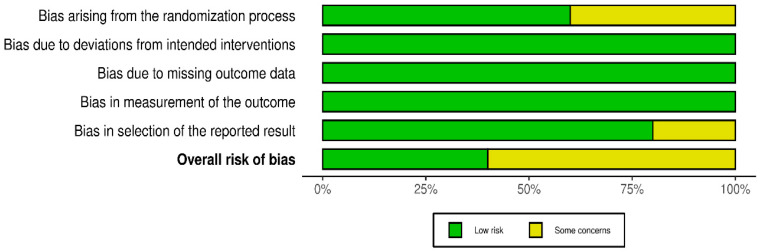
A summary plot of the RoB 2 tool for the included studies.

The studies included in this systematic review are generally of high methodological quality. According to the assessment using the RoB 2 tool, two of the five studies had a low risk of bias in all five evaluated domains, while the other three studies had a level of “some concerns” rating in one domain. Details of the assessment are presented in Figure 2 and Figure 3.

### 3.3. GRADE Assessment

To better understand the overall confidence in the evidence and the reliability of the findings, we conducted a GRADE assessment for all included studies.

The results of the GRADE assessment for the included studies are summarized and highlighted in Table 4.

The GRADE assessment of the included studies shows that although there is evidence of positive effects of therapy, the certainty of the evidence is low due to the small number of studies and participants, as well as the methodological limitations reported. Consequently, the results should be interpreted with caution, and further high-quality research is needed to confirm these effects.

### 3.4. Detailed Description of the Studies

The assessment conducted using the RoB 2 tool revealed that, of the five included studies, three had a moderate risk of bias (“some concerns”), and two were assessed as having a low risk. Overall, the methodological quality of the studies is acceptable, but the results must be interpreted with caution, given the identified limitations.

According to GRADE, the level of confidence in the available evidence is low, highlighting the need for further research.

Table 5 provides an overview of the most important aspects of each study: the author, the site of the therapy application, the characteristics of the focal vibration, the objectives pursued, and the follow-up period of the participants. The robustness of findings was qualitatively assessed by considering differences in study design, population characteristics, intervention types, and outcome measures across the included studies. The certainty of the evidence was considered based on the overall quality and consistency of the included studies.

To facilitate the interpretation and comparability of the results, a summary of participant characteristics (age, sex, disease stage, and medication), safety indicators (adverse events and adherence to the intervention), and intervention-specific parameters (intensity of therapies, tools used, and therapist experience) is presented in Table 6. This approach allows for concise integration of essential information and provides an overview necessary for contextualizing the results.

In a randomized, double-blind, double-dummy study conducted by Volpe et al. [24], the efficacy of a balance rehabilitation program using a wearable proprioceptive stabilizer was evaluated in patients with PD. The 40 enrolled patients were randomly assigned to two groups (active device vs. placebo), both of which underwent the same physiotherapy protocol for two months. At T1, in the context of visual deprivation, the instrumented functional reach test showed significant improvements in sway area and limits of stability exclusively in the active device group, suggesting enhanced proprioceptive-vestibular integration. The placebo group exhibited only transient effects, with no significant change in fall rate. The final results indicate a potential synergistic effect between the FMV and balance rehabilitation exercises. The study supports the clinical potential of this intervention and highlights the need for further research in larger cohorts [24].

A randomized controlled trial conducted by F. Camerota et al. [25] investigated the effects of repetitive focal muscle vibration (r-fMV) on gait parameters in patients with PD. The 20 participants were divided into two groups: one receiving real r-fMV and the other receiving a placebo treatment. The group receiving r-fMV demonstrated significant improvements in several biomechanical gait parameters. Gait speed increased significantly at 24 h and one week after treatment (*p* = 0.036 and *p* = 0.02, respectively), and step length and swing velocity were significantly higher at all three post-treatment evaluations (T1, T2, T3; *p* < 0.05). Other variables (step width, cadence, stance time, and double support) did not show significant changes. The benefit of average gait speed and step length persisted for up to three weeks after intervention. The placebo group showed no significant changes in these parameters. The study suggests that r-fMV may stimulate neural circuits involved in locomotion by facilitating sensorimotor integration at the central nervous system level [25].

In a double-blind, double-dummy, randomized crossover study, Peppe et al. [26] analyzed the effect of focal proprioceptive stimulation on gait parameters in patients with PD. The 40 participants were randomly assigned to receive either active treatment or a placebo for 8 weeks. After a 4-week wash-out period, the interventions were reversed for another 8 weeks. The primary objective was to assess changes in spatiotemporal gait parameters (speed, step length, double support), measured using a 3D stereophotogrammetric system. Following the active treatment, significant increases in average gait speed and step length, as well as reductions in double support duration, were observed bilaterally. No relevant changes were seen in the placebo group. Patients at Hoehn & Yahr stage 3 showed more pronounced improvements, suggesting enhanced proprioceptive responsiveness in more advanced stages of the disease. No carry-over effect was identified between treatment phases (T0 and T2). Despite the limited sample size, the findings provide promising evidence regarding the potential of the device to improve axial symptoms and gait parameters in patients with PD [26].

In a randomized, crossover, double-dummy, controlled study, Romanato et al. [27] investigated the effects of focal mechanical vibrations on gait biomechanics and postural control in patients with PD. The 24 participants were randomly assigned to receive either active or placebo treatment for 8 weeks, followed by a 4-week wash-out period, after which the interventions were reversed for another 8 weeks. The primary objective of the study was to evaluate biomechanical changes in gait through 3D stereophotogrammetric analysis of lower limb joint kinematics, and static postural control was assessed using the instrumented Romberg test. The results showed that active treatment led to significant increases in step length and reductions in step time. In the Romberg test, active treatment reduced postural effort and shifted spectral parameters toward frequencies typical of healthy subjects, especially under eyes-closed conditions, suggesting improved proprioceptive-vestibular integration. This study provides evidence supporting the efficacy of focal mechanical vibration in improving gait biomechanics and postural stability in patients with PD, even in the absence of concomitant rehabilitation therapy [27].

In a double-blind, randomized, crossover clinical trial, Spolaor et al. [28] investigated the effects of focal proprioceptive stimulation on postural control and muscle activity in patients with PD. Forty participants underwent two treatment phases (active and placebo), each lasting 8 weeks and separated by a 4-week wash-out period. Of the 25 patients selected for surface electromyography analysis, 20 completed the full protocol. Assessments included 3D stereophotogrammetric gait analysis synchronized with force platforms and bilateral 8-channel surface EMG. Focal proprioceptive stimulation with Equistasi may support postural control and muscle activity in patients with PD. This study indicated that focal mechanical vibrations could enhance motor control strategies in patients with PD, improving both balance and gait control [28].

Most of the included studies [25,26,27,28] reported significant improvements in gait parameters (speed, stride length, reduced double-support time) and postural control, supporting the hypothesis that focal or mechanical proprioceptive stimulation can facilitate sensorimotor integration in patients with Parkinson’s disease.

The results obtained by Volpe and Romanato [24,27] show that these interventions not only influence the biomechanical parameters of gait, but also complex sensory integration processes (proprioceptive and vestibular). This is essential, given the role of sensory deficits in postural instability in PD.

Camerota [25] showed that the benefits on speed and stride length can be maintained up to three weeks post-intervention, suggesting a potential temporary reconfiguration of the neural circuits involved in locomotor control. However, the persistence of long-term effects remains unclear and requires further investigation.

Peppe et al. [26] showed that patients in Hoehn & Yahr stage 3 responded more favorably to treatment, suggesting that proprioceptive stimulation interventions may be particularly useful in the intermediate-advanced stages of the disease, where postural and axial deficits become dominant.

In all studies included in the analysis [24,25,26,27,28], placebo groups did not show significant improvements, which supports the specificity of the effect of the stimulation mechanism and reduces the likelihood of a simple psychological influence on the results.

Overall, the studies [24,25,26,27,28] converge towards the idea that muscle vibration is a safe and effective intervention with effects on gait, posture, and motor control in patients with PD. However, methodological limitations (small number of participants, short follow-up periods, heterogeneity of protocols) require validation of these results in larger, well-designed clinical trials.

Potential sources of heterogeneity of the studies were considered by examining differences in study characteristics, interventions, and outcomes, without formal statistical testing.

Assessments of confidence in the evidence were conducted by considering the overall patterns and reliability of the study results.

## 4. Discussion

### 4.1. Parkinson’s Disease in Light of Recent Evidence

In PD, the movement disorder involves various causes, affecting both the central and peripheral nervous systems. It is the most common neurological disease, ranking second overall, with patients over the age of 60 accounting for 1% of the affected population [21,29]. This neurological condition is progressive, caused by a dopamine deficit that leads to tremor, bradykinesia, rigidity, and gait impairment [30].

Postural control disorders frequently lead to falls, representing a significant risk for patients with PD; as a result, activities of daily living are significantly limited. Compared to healthy individuals, patients with PD exhibit reduced limits of stability in static upright posture [31]. Studies show that 90% of all patients with PD will experience a fall at some point in their lifetime [32].

Studies in the literature define camptocormia as a postural disorder characterized by marked flexion of the thoracolumbar spine, present in standing and walking, with a reported prevalence in PD between 3% and 18%. This condition differs from common kyphoscoliosis in that it is partially relieved by posterior support, but recurs with prolonged maintenance of the upright position. Regarding etiopathogenesis, several mechanisms have been proposed, including basal ganglia dysfunction with hyperactivity of flexor muscles, paraspinal myopathy, axial dystonia, or adverse effects of drug treatment. In some cases, camptocormia is considered a compensatory mechanism to maintain stability, but it is frequently associated with gait disorders, low back pain, dyspnea, and dysphagia [33].

Among patients with PD, the perception of limb position and passive movements is impaired, leading to difficulties in force regulation and weight perception. These proprioceptive and haptic deficits may contribute to altered motor control and increased reliance on visual cues. Deep brain stimulation (DBS) of the subthalamic nucleus appears to partially improve these functions, possibly by reducing neuronal noise involved in kinesthetic signal processing. Although dopaminergic treatment remains essential, its effectiveness diminishes over time, and medication resistance may develop. In this context, physiotherapy, particularly proprioceptive training, becomes a crucial component, aiming to improve mobility and quality of life [34].

Invasive alternatives to pharmacological treatments include stereotactic thalamotomy, spinal cord stimulation (SCS), DBS, and continuous intestinal infusion of levodopa-carbidopa or apomorphine. Among these, DBS is considered the surgical gold standard in PD. SCS shows promising results in managing pain associated with this condition, while intestinal levodopa gel therapy helps reduce motor fluctuations and improve quality of life. However, invasive procedures carry significant risks, such as intracranial hemorrhage in the case of DBS, which may limit their applicability in certain patients. Consequently, a new non-invasive therapeutic approach is being explored through the use of various medical devices [35].

### 4.2. Focal Vibration Therapy in PD—Evidence and Perspectives from the Literature

In PD, rehabilitation is considered an essential adjunct to pharmacological and surgical treatments to maximize functionality and reduce the risk of secondary complications. While rehabilitation strategies were initially based predominantly on empirical experience, increasing evidence now supports that exercise-induced neural plasticity is the central mechanism of the effects obtained with physiotherapy. Recent meta-analyses indicate that rehabilitation interventions can generate significant clinical benefits, particularly with regard to gait and balance [36].

Rehabilitative interventions should be regarded as a fundamental component of the comprehensive management of PD, starting from diagnosis and continuing through the advanced stages. Greater efforts are needed to educate and raise awareness about the benefits of these services for people with PD, along with the promotion of further evidence-based scientific research [37].

Restoring motor function by modulating proprioceptive signals using non-specific repetitive muscle vibrations applied focally to individual muscles represents an innovative approach, different from exclusive training of muscle function. This perspective is supported by the results obtained in healthy individuals, where improvements in motor performance have been observed. FMV involves the application of micro-stretching and shortening sequences to the target muscle, and the repetition of these stimulations can generate a rapid and lasting increase in motility [38].

Vibration therapy induces a global neuromuscular activation and has been used in rehabilitation since 1969, when Hagbarth and Eklund discovered that vibration elicits a contraction of the agonist muscle [39].

FMV is an emerging technique aimed at improving balance and motor control in a range of neurological conditions. It involves delivering a low-amplitude, high-frequency vibratory stimulus to a specific muscle via a mechanical device. The application of FMV in rehabilitation is relatively recent, yet it shows considerable potential as an effective means of enhancing rehabilitation outcomes. In recent years, its use has grown due to several advantages over conventional rehabilitation methods, including greater patient adherence to treatment protocols [9].

Equistasi is a portable, small-sized technological solution with wireless connectivity, designed to support patients with PD who experience episodes of freezing of gait (FOG). Through an integrated algorithm, it enables rapid and accurate identification of motor blocks and provides an external sensory stimulus intended to facilitate the resumption of movement [40].

Approved by the Ministry of Health in 2020, Equistasi uses high-frequency focal vibration technology (~9000 Hz). The device is thermoactive and automatically generates mechanical vibrations upon contact with the skin. With its small size (1 × 2 cm) and minimal weight (0.17 g), it can be worn during daily activities or recovery sessions. It is reusable and can be applied to multiple anatomical regions, depending on the medical indication. According to the manufacturer’s specifications, there is no shelf life determined for its use [28].

The application of the device to a muscle tendon is believed to modulate the activity of Golgi tendon organs, which are essential components of the proprioceptive system. Randomized clinical studies have demonstrated its effectiveness in movement disorders such as PD and multiple sclerosis sequelae, showing improvements in balance, gait, and overall motor function. These effects are thought to result from an optimization of proprioceptive feedback, although the underlying physiological mechanisms remain insufficiently understood [41].

In addition to the demonstrated benefits on motor control and balance, Equistasi may also have potential analgesic effects, as suggested by studies investigating the impact of focal microvibration on chronic musculoskeletal pain. The results indicate a significant reduction in pain intensity, an improvement in quality of life, and even a possible decrease in the need for analgesic medication. These preliminary findings suggest an expansion of the device’s clinical indications; however, further research is needed to clarify the underlying mechanisms and the long-term sustainability of its effects [42].

Numerous studies have investigated the effectiveness of the Equistasi device on gait and balance in patients with PD. The results obtained from various studies open relevant perspectives for further research. Alessandro M. De Nunzio et al. reported that vibration applied to the paravertebral muscles increases gait speed in patients with PD, with comparable effects observed in healthy subjects. It was concluded that the method could be useful for improving gait, but further studies are needed to evaluate the potential placebo effect and the effectiveness of the device in patients during periods without medication, when motor symptoms reappear or worsen [43].

FMV plays a crucial role in reducing spasticity and enhancing gait and balance, even in patients who have suffered a stroke. Conversely, vibration therapy has not proven effective in reducing spasticity in multiple sclerosis and cerebral palsy. FMV appears to be more effective when applied to non-spastic antagonist muscles to induce reciprocal inhibition of the spastic ones [44].

The study by Spolaor et al. suggests that FMV applied through the Equistasi device can facilitate motor control strategies in patients with PD, thereby contributing to improvements in both balance and gait mechanics [28].

Research conducted by Alashram et al. confirms the benefits of focal proprioceptive stimulation with the Equistasi device in improving gait and postural stability in patients with PD. The therapy was reported to be safe, well-tolerated, and promising in optimizing motor functions [45].

The percentage assessment of the phases of the gait cycle, carried out in a study, revealed a significant reduction in the duration of the stance, swing, and double support phases for both lower limbs when the device was activated. In contrast, when the device was deactivated, the observed changes did not reach statistical significance, except for a non-significant reduction in double support on the left side [26].

A retrospective, observational, long-term follow-up study showed that the use of FVT as part of a rehabilitation protocol was not associated with any significant adverse events. These findings support the safety and tolerability of the Equistasi device in patients with PD. It was concluded that patients who followed a combined protocol of focal vibration and rehabilitation experienced a significant reduction in fall frequency, while their daily levodopa equivalent dose remained stable, indicating good tolerability of the intervention [46].

Several authors have investigated the possible mechanisms through which focal proprioceptive stimulation delivered by Equistasi may influence gait in patients with PD and other neurological disorders. A study conducted in 2019 concluded that the improvement in gait parameters—such as average speed, step length, percentage of stance phase, and double support time—is attributable to proprioceptive stimulation via focal mechanical vibration. Notably, the same study reported a more pronounced improvement in symptoms among PD patients at Hoehn & Yahr stage 3, which may reflect an enhanced capacity to integrate proprioceptive stimuli in the moderate stages of the disease [26].

Volpe D. explained the physiological mechanism of this therapy as being related to the endings of muscle spindles, which are highly sensitive to external vibrations. Under such conditions, they transmit proprioceptive input to the central nervous system, which can modulate the excitability of spinal reflexes and muscle responses to postural disturbances [24].

Another mechanism proposed in the scientific literature relates to the vibration-induced synchronization of motor units, which may influence muscular performance. It is suggested that this forced synchronization leads to a decrease in contraction efficiency, possibly by affecting the excitation–contraction coupling process. This phenomenon, observed in the context of the tonic vibration reflex, may contribute to changes in muscle tone and the onset of muscle fatigability under prolonged stimulation conditions [47].

Cruciani et al. hypothesized that the focal vibrations generated by the Equistasi device may influence cortical somatosensory processing. The authors observed a reduction in the late component of high-frequency oscillations (HFOs), which could indicate a restoration of the balance between sensory and motor circuits. This type of cortical modulation may support more efficient sensorimotor integration, which is relevant in the context of rehabilitation for PD and other neurological disorders, such as dystonia [41].

As found in the study by Romanato M. et al., the beneficial effects of FMV can be observed even in patients who do not follow a concomitant physiotherapy program. The results revealed a significant increase in step length and a decrease in their duration, along with a reduction in the amplitude of movements in the sagittal plane at the level of the trunk and ankle joints, with values close to those found in healthy subjects [27].

The recent review by L. Moggio aimed to evaluate the role of vibration therapy in the rehabilitation of neurological conditions. Both whole-body vibration (WBV) and FMV appear to have a significant impact in reducing spasticity and improving gait, balance, and motor function [44].

Because muscle strength is essential in maintaining daily autonomy and athletic performance [48], it is important that FMV intervention is safe and well-tolerated in people with neurological disorders [49,50]. Thus, FMV can be considered a valuable method that deserves to be included in the complex management of these conditions [51].

According to the review by Luigi Fattorini, focused vibration is an effective stimulus, capable of improving motor skills by optimizing agonist/antagonist interaction and reorganizing central and segmental nerve pathways. The studies analyzed reported long-lasting positive motor changes, without adverse effects, and the most effective protocol seems to involve a frequency of ~100 Hz, applied 3–5 days per week and on contracted muscles [52].

The application of non-specific repetitive muscle vibrations focused on individual muscles represents an important perspective in the restoration of motor functions, because it acts on proprioceptive signals and not only on muscle function. Studies suggest that this therapy can counteract motor deficits, leading to an early and rapid recovery of motor performance. The effectiveness has been demonstrated in various clinical conditions, with immediate motor improvements obtained without straining the joints [38].

Localized muscle vibration therapy is a promising intervention in the rehabilitation of several neurological pathologies, including post-stroke, demonstrating efficacy in reducing spasticity and improving motor recovery of both upper and lower limbs. Studies suggest that it is feasible and safe, and can be successfully integrated into conventional neurorehabilitation programs, enhancing the effects of classical physical therapy [53].

Luigi Fattorini, in 2025 [8], conducted a review of focal muscle vibration, highlighting that this intervention can reduce spasticity and improve motor function. The analysis included 20 studies, most of which focused on post-stroke spasticity, but also on conditions such as cerebral palsy, multiple sclerosis, or Minamata syndrome, using functional scales, digital analyses, and electrophysiological assessments. In 19 studies, the therapy produced positive and significant motor effects, while only one study reported nonsignificant results [8].

Another recent review on neurological rehabilitation examined the use of whole-body vibration (WBV) and FMV compared with placebo, sham, or other types of exercise in children and adults with motor impairments and disabilities. The review included 16 systematic reviews of good methodological quality and found that, in stroke patients, WBV can improve gait and balance, while FMV is more effective in reducing spasticity. In patients with multiple sclerosis and cerebral palsy, there is no consistent evidence of significant improvements in motor function [44].

Classical FVT protocols generally use frequencies ranging from 100 to 180 Hz [25,52,54], while the Equistasi device is characterized by the generation of vibrations that can reach up to approximately 9000 Hz [24,26,27,28]. In the specialized literature, studies that used Equistasi [24,26,27,28] described this intervention under the name of FVT, which is why we included these works in this review. However, it should be noted that this methodological diversity highlights the need for future research to differentiate the specific effects of each type of vibration and the physiological mechanisms involved.

### 4.3. Evidence from the Studies Analyzed

All studies reviewed included patients with Hoehn & Yahr stages 2–3 Parkinson’s disease, suggesting that vibration interventions were tested primarily in people with moderate motor impairment [24,25,26,27,28].

The mean age was around 65–70 years [24,25,27,28] and the gender was relatively balanced (approximately 40–50% male). This showed that the target population was homogeneous and the results can be compared between studies.

All patients in the 5 studies [24,25,26,27,28] were receiving levodopa, with doses expressed as LEDD (Levodopa Equivalent Daily Dose). This is important because the effects of vibration therapy must be interpreted in the context of dopaminergic therapy, which remains the gold standard.

Most studies reported no major adverse events (Volpe [24], Peppe [26], Romanato [27]). Camerota [25] mentioned the intervention as “potentially safe and feasible,” but without detailed data. Spolaor [28] did not explicitly report adverse events. Vibration therapy appears safe and well-tolerated in patients with Parkinson’s. However, not all studies rigorously report safety, which limits comparison.

Regarding the intervention parameters, there is a lack of uniformity and complete reporting of vibration parameters, which makes it difficult to reproduce the protocols.

The evaluation was multimodal, integrating both validated clinical scales and instrumental measurements. All studies used the UPDRS-III (or MDS-UPDRS III), and some added scales for balance, fear of falling, and quality of life (BBS, ABC, FES, TUG, PDQ-39, number of falls—Volpe [24], ABC, PDQ-39, rate of falls—Romanato [27], ABC, PDQ-39—Spolaor [28]). In parallel, objective methods such as 3D gait analysis (Camerota [25], Peppe [26]), posturography (Romanato [27], Spolaor [28]), and sEMG (Spolaor [28]) were used. This combination provides a more complete picture of the effects of vibration therapy.

Non-motor symptoms—including pain, cognitive impairment, and fatigue—were not adequately addressed in the studies reviewed [24,25,26,27,28]. Regarding quality of life, although it has been evaluated as a parameter in several studies, the results have not been reported, except for the study conducted by Volpe [24]. At the T1 assessment, a significant improvement in quality of life was observed, according to PDQ-39 scores. At T2, this improvement was maintained, indicating that the intervention had a positive and sustained effect on the perception of quality of life in the patients included in the study [24].

All studies used blinded therapists [24,25,26,27,28], which strengthens the validity of the results. In some cases, a “double-blind, double-dummy” design was mentioned, which shows good methodological quality [26,27].

The protocols used in the five studies analyzed [24,25,26,27,28] varied significantly in terms of the location of stimulation application, the frequency of sessions, the duration of each intervention, and the post-treatment follow-up period, aspects that largely explain the heterogeneity of the reported results. Currently, there are no standardized and unanimously accepted protocols, which makes it difficult to directly compare the effects and generalize the conclusions.

Analysis of the included studies [24,25,26,27,28] reveals that the follow-up period was relatively short, focusing mainly on the immediate and short-term effects of the therapy used. Most studies performed assessments at baseline (T0), immediately after the completion of the treatment period (T1), and, in some cases, after a short wash-out period or at the beginning of a second treatment phase (T2–T3). For example, Volpe [24] performed assessments at baseline, immediately after two months of therapy, and two months after its completion. In contrast, the studies by Peppe [26], Romanato [27], and Spolaor [28] included two 8-week treatment cycles separated by a 4-week wash-out interval. Camerota [25] performed assessments 24 h, 1 week, and 3 weeks after the last session.

This follow-up structure allows for the observation of immediate and short-term effects, but does not provide information on the durability of long-term effects or on possible adaptations of the body to vibration therapy over time. Thus, the results obtained indicate the effectiveness of the intervention in the short term, but limit the possibility of concluding the long-term impact, the need for continued treatment, or possible cumulative or late effects.

In conclusion, the existing literature emphasizes the short-term effectiveness of vibration therapy, but also highlights the need for studies with extended follow-up to assess the stability and durability of benefits, as well as the long-term safety of this intervention. Only through a longitudinal evaluation can the clinical relevance and applicability of these interventions be truly determined.

In the case of FMV in patients with PD, practical considerations such as patient adherence, ease of application at home, cost-effectiveness, and comparison with standard rehabilitation programs have not been sufficiently addressed in the existing literature. However, these aspects are essential for assessing the feasibility and clinical adoption of the intervention, as even significant motor benefits may be limited if practical implementation proves difficult or costly.

### 4.4. The Strengths of the Study

This review was conducted using the PRISMA methodology, which ensured the transparency of the selection process and the reproducibility of the results. The extensive search was performed in five major and relevant medical databases, thus increasing the coverage of the available literature and reducing the risk of omitting important studies. Clear inclusion criteria, focusing on controlled studies (parallel or crossover), contributed to strengthening the quality of the synthesized evidence, and the focus on studies with a control group increases confidence in the validity of the conclusions formulated. In addition, the critical analysis of methodological quality was performed by applying the ROB 2 scale to assess the risk of bias, ensuring a rigorous interpretation of the results. Through this systematic and rigorous approach, the review provides an up-to-date and relevant synthesis of FMV in PD, representing a solid starting point for future research.

### 4.5. Limitations of the Study

In the present analysis, the interpretation of the results is influenced by several methodological limitations. First, the small number of included studies (n = 5) limits the possibility of formulating generalizable conclusions. Also, the exclusive inclusion of articles published in English introduces the possibility of omitting relevant data published in other languages. Most of the analyzed studies had relatively small sample sizes, which reduces statistical power. The selection criterion by which only controlled studies (parallel or crossover) were included, although it increased methodological rigor, led to the exclusion of observational studies that could have provided additional information on the applicability and safety of the intervention.

In addition, the heterogeneity of vibration therapy parameters (frequency, amplitude, duration, location) limits direct comparability between studies. Also, the high variability of patient characteristics, including differences in disease severity, concomitant treatments, and the presence of comorbidities, may influence the response to therapy and reduce the uniform applicability of the conclusions.

An important limitation of the studies reviewed is the relatively short follow-up period, which allowed for the evaluation of the effects of vibration therapy only in the short term. This prevents the assessment of the durability of benefits, possible cumulative effects, and long-term safety, which reduces the generalizability of the conclusions.

### 4.6. Future Research Directions

All the aspects listed under “Limitations of this study” emphasize the need for future larger, multicenter studies with bigger samples, standardized designs, and extended follow-up periods to confirm and clarify the role of FMV in PD.

Given that the progression of PD is not linear, often showing variable and sometimes accelerated rates of decline in the early stages, further research is needed in different stages of the disease to identify subgroups of patients who may benefit most from this type of intervention.

To advance the understanding and application of FMV in PD, multicenter studies with large sample sizes and standardized protocols are needed, allowing for the comparability of results. Investigation of long-term effects and safety monitoring is essential to assess the durability of benefits. It is important to include objective measures and biomarkers for an accurate assessment of therapeutic response, as well as studies that analyze the combination of therapy with other treatments.

Because the effects of FMV on non-motor symptoms of PD are underreported in most of the included studies, this limits the full understanding of the impact of the intervention and suggests the need for greater attention to these outcomes in future research.

To ensure the comparability and relevance of the results, future studies could adopt a minimum panel of standardized instruments for assessing non-motor symptoms, such as the Parkinson’s Disease Questionnaire (PDQ-8) or PDQ-39, which includes quality of life, depression, and sleep disturbance scales. This approach would facilitate the synthesis of results across studies and allow a more complete assessment of the effects of the therapy.

It is also recommended to implement a standardized framework for reporting adverse events, so that patient safety is systematically documented and comparable across studies. This would contribute to the transparency and validity of conclusions regarding the tolerability of the therapy.

## 5. Conclusions

The efficacy of FVT remains potential, but cannot be confirmed with certainty due to the small number of studies, short follow-up periods, variation in protocols applied, insufficient consideration of practical considerations (adherence, home use, cost-effectiveness), and limited assessment of non-motor symptoms.

Nevertheless, FVT has demonstrated promising benefits and an innovative character in the management of motor symptoms in patients with PD. It has been shown to exert a beneficial effect on some essential parameters in patients with PD by improving postural instability, optimizing gait, and improving balance. These advances translate into an increased ability of patients to control their posture and maintain stability during movement, thus reducing the risk of falls and associated complications. Studies conclude that this therapy significantly contributes to these essential aspects in maintaining patients’ independence and quality of life.

In addition, this non-invasive method offers valuable therapeutic potential, complementing traditional rehabilitation strategies, and can be easily integrated into multidisciplinary programs dedicated to Parkinson’s patients.

These results support the integration of FVT as a valuable component in complex rehabilitation programs for PD. However, further research is needed to establish optimal application parameters and to evaluate the long-term beneficial effects of FVT.

## Figures and Tables

**Table 1:** The combinations of keywords employed in the searches conducted across international databases.

	PubMed	PEDro	Science Direct	Cochrane Library	Web of Science	Total
Focal vibration AND Parkinson’s	27	2	2203	9	31	2272
Focal vibration AND Parkinson’s disease	24	2	2000	9	26	2061
Focal muscle vibration AND Parkinson’s	12	2	1655	7	20	1696
Focal muscle vibration AND Parkinson’s disease	9	2	1612	7	17	1647
Focal vibration therapy AND Parkinson’s	16	0	1436	6	6	1464
Focal vibration therapy AND Parkinson’s disease	15	0	1416	6	5	1442
FVT AND Parkinson	1	0	47	0	1	49
FVT AND Parkinson’s disease	1	0	37	0	1	39
Total	105	8	10,406	44	107	10,670

**Table 2:** Summary of inclusion and exclusion criteria according to the PICOS framework.

PICOS	*The Inclusion Criteria*	*The Exclusion Criteria*
**Population (P)**	PD diagnosis	Patients with another medical condition, besides PD; animal studies or preclinical studies without clinical validation
**Intervention (I)**	Studies that used focal muscle vibration therapy	Other therapies used, apart from focal muscle vibration therapy
**Comparison (C)**	Control group studies or crossover studies	Studies that do not have a control group
**Outcomes (O)**	Improvement of symptoms: motor function, functional capacity, quality of life, reduction in disability	Results not relevant for focal muscle vibration therapy or not presented
**Study design (S)**	Original studies (randomized controlled trials, case–control studies, cohort studies, observational studies, trials)	Systematic reviews, meta-analyses, books, book chapters, editorials, conference abstracts, and notes

**Table 3:** Summary of included studies.

Author’s Name	Year of Publication	Study Design	Sample Size
Volpe et al. [24]	2014	A double-blind, double-dummy, parallel group, randomized controlled trial, placebo	40
Camerota et al. [25]	2016	A single-blind, parallel-group study design, Pilot Randomized, Controlled Trial, placebo	20
Peppe et al. [26]	2019	A multicentric randomized, double-blind crossover study, placebo	40
Romanato et al. [27]	2021	Crossover double-dummy, double-blind, randomized, controlled study, placebo	24
Spolaor et al. [28]	2021	A multicenter, randomized, double-blinded crossover study, placebo	20

**Table 4:** GRADE Assessment.

Outcomes	Anticipated Absolute Effects * (95% CI)		Relative Effect (95% CI)	Number of Participants (Studies)	Certainty of the Evidence (GRADE)
	Risk with [Comparison]	Risk with [Intervention]			
**Effectiveness of the balance training program**	Static posturography parameters have not changed. No significant effect was found for the fall rate.	Improving clinical variables assessing self-confidence, balance, and disability. Having an overall positive impact on health-related quality of life. A significant effect was found for the fall rate.	-	40 (1 study)	Low ^a,b,c^ ⊕⊕⊝⊝
**Effects on gait**	No significant differences in all the gait variables.	Gait improvement as a result of increased walking velocity and stride length.	-	60 (2 studies)	Low ^d,e,f^ ⊕⊕⊝⊝
**Coupling between balance and lower limb joints kinematics**	Initial kinematic maintenance.	Improvements in trunk flexion, extension, and ankle dorsi-plantar flexion. Balance assessment—improvements at the frequencies corresponding to the vestibular system.	-	24 (1 study)	Low ^g,h,i^ ⊕⊕⊝⊝
**Relationship between muscular activity and postural control changes**	Increased surface electromyography activity was observed in both gastrocnemius lateralis and biceps femoris.	A reduction in the postural efforts and the peripheral vestibular disorders. Surface electromyography revealed a change in the motor control after the treatment. Increase in stride length.	-	20 (1 study)	Low ^j,k,l^ ⊕⊕⊝⊝

Explanations: a. Small sample size (n = 40) limits the precision of the effect estimate. b. Only one study reported fall rate, limiting the ability to assess consistency across studies. c. Some concerns regarding blinding and allocation concealment; risk of performance bias cannot be excluded. d. Two studies included (n = 60 total), but sample sizes are small and confidence intervals are not fully understood, leading to imprecision. e. Variability in gait improvements across studies; results not fully consistent. f. Potential publication bias suspected due to predominance of positive findings and lack of unpublished negative results. g. Only one study available (n = 24); inconsistency could not be assessed. h. Small sample size and wide variability in kinematic measures contribute to very serious imprecision. i. The study design is limited, increasing the risk of selection and performance bias. j. Only one study (n = 20); limited precision due to small sample size and wide confidence intervals. k. Positive effects reported, but replication is lacking, so certainty remains low. l. Some concerns about the risk of bias in electromyography measurements; blinding is unclear. The corresponding risk * (and its 95% confidence interval) is based on the assumed risk in the intervention group and the relative effect of the intervention (and its 95% CI). Certainty in an estimate of effect across those considerations: Low ⊕⊕◯◯.

**Table 5:** Detailed description of included studies.

Author’s Name/Country	The Site of the Therapy Application	Intervention	The Main Objective	Follow-Up Period
Volpe et al. [24] Italy	Each patient wore 3 Equistasi devices, applied over the 7th cervical vertebra and on each soleus muscle tendons.	A 60-min physiotherapy session, five days a week, for two months. During the first three weeks of rehabilitation, both groups wore the devices six days per week for: 60 min per day in the first week, 120 min in the second, and 180 min in the third. From the fourth week, the devices were worn five days a week for four hours each day.	Effectiveness of a wearable proprioceptive stabilizer (Equistasi) for the rehabilitation of postural instability.	Baseline measurements (T0) were collected within one week before enrollment. The second evaluation (T1) was conducted within one week after completing the two-month therapy period The final assessment (T2) took place two months after T1.
Camerota et al. [25] Italy	The transducer was placed bilaterally on the quadriceps tendon near the insertion of the rectus femoris, approximately 2 cm from the medial border of the patella, as well as on the lumbar paraspinal muscles.	For each muscle group, r-FMV was administered in three 10-min sessions, separated by 1-min intervals, resulting in a total application time of 60 min. This protocol was repeated over three consecutive days to induce cumulative after-effects.	Beneficial effects of repetitive sessions of r-fMV on gait (using the Cro system).	A GA evaluation was done before r-fMV (T0) and 24 h (T1), 1 week (T2), and 3 weeks (T3) after the last session of r-fMV.
Peppe et al. [26] Italy	The three plaques were placed on the skin as follows: one over the seventh cervical vertebra and one on the tendon of each soleus muscle.	The device was worn for 6 days/week, starting with 1 h/day in the first week and increasing by 1 h weekly until reaching 4 h/day by week 4. This duration was then maintained for another 4 weeks, worn 5 days/week. At the end of the first 8-week period (T1), patients were evaluated for primary and secondary endpoints, followed by a 4-week wash-out phase. After re-evaluation (T2), patients began the crossover phase with the alternate kit.	This study aims to evaluate the clinical effects of proprioceptive system modulation on gait performance in individuals with PD using the Equistasi device.	The baseline assessment (T0) was conducted before enrollment. The second evaluation (T1) took place at the end of the first 8-week treatment period. The third assessment (T2) was performed at the start of the second 8-week treatment phase, following a 4-week wash-out period. The final evaluation (T3) occurred at the end of the second 8-week treatment period.
Romanato et al. [27] Italy	The device was applied to the skin in the following locations: one over the seventh cervical vertebra and one on each soleus muscle.	Participants underwent two 8-week treatment phases (active/placebo), separated by a 4-week wash-out. The device was worn 6 days/week in the first week for 1 h/day, increasing to 4 h/day by week 4, then maintained at 4 h/day, 5 days/week, for the remaining 4 weeks.	The study aimed to investigate the effects of the Equitasi device on gait and balance.	T0: Baseline assessment conducted before enrollment; T1: Second evaluation at the end of the first 8-week treatment period; T2: Third evaluation following the 4-week wash-out period, marking the start of the second treatment phase; T3: Final assessment at the end of the second 8-week treatment period.
Spolaor et al. [28] Italy	The device was applied on the skin as follows: One over the 7th cervical vertebra and two over each soleus muscle.	Participants received treatment for 8 weeks, without any additional rehabilitation procedures. After a 4-week wash-out period, they crossed over to the other treatment for another 8 weeks. During the first week, the device was worn for 1 h/day, 6 days/week. Over the next 3 weeks, the wearing time increased by 1 h per week, reaching 4 h/day by week 4. In the final 4 weeks, the device was worn for 4 h/day, 5 days/week.	This study aimed to examine how the Equistasi wearable device influences the relationship between muscle activity and changes in postural control.	Patients were evaluated at four time points: (T0) baseline before enrollment; (T1) after the first 8-week treatment period; (T2) following the 4-week wash-out period, which marked the start of the second treatment phase; and (T3) after the second 8-week treatment period.

Legend: r-fMV = Repetitive sessions of focal muscle vibration; fMV = Focal muscle vibration; GA = Gait analysis; PD = Parkinson’s Disease.

**Table 6:** Participant characteristics, intervention parameters, and safety indicators.

Author’s Name	Age	Sex	Disease Stage	Disease Duration	Medication –ON State	Adverse Events	Treatment Adherence	Intensity of Therapies	Tools Used	Therapists’ Experience
Volpe et al. [24]	AG = 66.5 (64.0; 78.0) PG = 69.5 (65.0; 73.8) *p* value = 0.947	AG = 7 M/13 F PG = 9 M/11 F *p* value = 0.747	Hoehn and Yahr stage: AG = 3.0 (3.0; 3.0) PG = 3.0 (2.0; 3.0) *p* value = 0.429	Active group: 6.0 (4.0; 10.8); Placebo group: 6.5 (4.0; 9.0)	Levodopa L-dopa LEDD: AG = 487.5 (315.0; 690.0) PG = 450.0 (293.8; 600.0) *p* value = 0.892	No	Successfully with high adherence in both groups	Equistasi- mechanical vibratory energy < 0.8 N, 9000 Hz	Clinical scales: UPDRS-II, UPDRS-III, BBS, ABC Scale, FES, TUG, PDQ-39; -Other measures: number of falls during the observation period.	Blinded hospital physiotherapists delivered the standardized rehabilitation and applied indistinguishable active/placebo devices
Camerota et al. [25]	AG = 67 ± 7.96 (53–74) PG = 65.5 ± 9.85 (48–79) *p* value = 0.74 Mean age: 64.85 ± 8.74 years	8 males and 12 females	Hoehn and Yahr stage: AG = 3 ± 0.45 (2–3) PG = 2.5 ± 0.39 (2–3) *p* value = 0.11	Study group: Median 8.0 5.57, Range 3.0–12.0; Control group: Median 7.5 3.70, Range 4.0–12.0	Levodopa L-DOPA LEDD: AG = 740 ± 53.75 (580–750) PG = 690 ± 116.14 (580–800)	None of the patients showed any side effects	Not reported	Cro System- low amplitude 200–500 μm and high frequency (100 Hz)	UPDRS-III, 3D gait analysis.	Standardized instructions; blinded to real/sham stimulation
Peppe et al. [26]	Age not reported; age at onset 60.3 ± 9.9 years	26 males and 14 females	Hoehn and Yahr stage: mean ± SD: 2.45 ± 0.50	Mean: 8.347, ±SD: 3.6	Levodopa L-DOPA LEDD: Therapy dose mean ± SD: 743.3 ± 293	No major adverse events during the study period were reported.	Not reported	Equistasi-frequency not reported	MDUPDRS III scores and 3D gait analysis (BTS system, stereophotogrammetry)	Therapists were blinded to treatment allocation (double-blind, double-dummy design)
Romanato et al. [27]	67.46 ± 10.27 years	15 males and 9 females	Hoehn and Yahr stage: 2.46 ± 0.51	Mean: 11.88, ±SD: 4.89	Levodopa L-DOPA LEDD: 757.14 ± 290.88	No adverse events or falls were recorded during the trial	Not reported (intervention described as tolerable)	Equistasi vibration intensity not reported	UPDRS-III, Posturography (incl. instrumented Romberg test), 3D gait analysis, ABC Scale, PDQ-39, and fall rate (last month)	Therapists were blinded to treatment allocation (double-blind, double-dummy design)
Spolaor et al. [28]	67.46 ± 10.27 years	13 males and 7 females	Hoehn and Yahr stage: 2.46 ± 0.51	Mean: 11.88, ±SD: 3.23	Levodopa L-DOPA LEDD: 757.14 ± 290.88	Not reported	Not reported	Equistasi- ~9000 Hz nanovibrations	UPDRS III, ABC scale, PDQ-39, Fall Rate (last month), sEMG; posturography (instrumented Romberg)	Therapists were blinded in a double-blind crossover design

Legend: AG = active group; PG = placebo group; UPDRS-II = The Unified Parkinson’s Disease Rating Scale II; UPDRS-III = The Unified Parkinson’s Disease Rating Scale III; BBS = Berg Balance Scale; ABC scale = Activities-specific Balance Confidence scale; FES = Falls Efficacy Scale; TUG = Time Up and Go test; PDQ-39 = Parkinson’s Disease Questionnaire—39; sEMG = surface electromyography; LEDD = Levodopa equivalent daily dose; MDUPDRS III = Movement disorder society-sponsored revision of the unified Parkinson’s disease rating scale III; M = Male; F = Female, SD = Standard deviation.

## Abbreviations

The following abbreviations are used in this manuscript (in alphabetical order):

ABC-scale	Activities-specific Balance Confidence scale
AD	Alzheimer’s disease
AG	Active group
BBS	Berg Balance Scale
DBS	Deep brain stimulation
F	Female
FES	Falls Efficacy Scale
FMV	Focal muscle vibration
FOG	Freezing of gait
FVT	Focal vibration therapy
GA	Gait analysis
GRADE	Grading of Recommendations Assessment, Development and Evaluation
HFOs	High-frequency oscillations
LEDD	Levodopa equivalent daily dose
M	Male
MDUPDRS III	Movement disorder society-sponsored revision of the Unified Parkinson’s disease rating scale III
PD	Parkinson’s disease
PDQ-8	Parkinson’s Disease Questionnaire
PDQ-39	Parkinson’s Disease Questionnaire—39
PEDro	Physiotherapy Evidence Database
PG	Placebo group
PRISMA	Preferred Reporting Items for Systematic Reviews and Meta-Analyses
SCS	Spinal cord stimulation
SD	Standard deviation
sEMG	Surface electromyography
R-fMV	Repetitive sessions of focal muscle vibration
RoB 2	Version 2 of the Cochrane risk-of-bias tool for randomized trials
TUG	Time Up and Go test
UPDRS-II	The Unified Parkinson’s Disease Rating Scale II
UPDRS-III	The Unified Parkinson’s Disease Rating Scale III
WBV	Whole-body vibration
